# Supplementary material for: Dynamic Displacement Vector Interacts with Tactile Localization
Source: Curr Biol. 2019 Feb 4;29(3):492–498.e3. doi: 10.1016/j.cub.2018.12.032 (PMC6370943; doi:10.1016/j.cub.2018.12.032)
Supplement: Document S1. Figure S1 and Tables S1 and S2 [file mmc1.pdf]

Current Biology, Volume *29*

Supplemental Information

Dynamic Displacement Vector Interacts with Tactile Localization

Lucile Dupin and Patrick Haggard

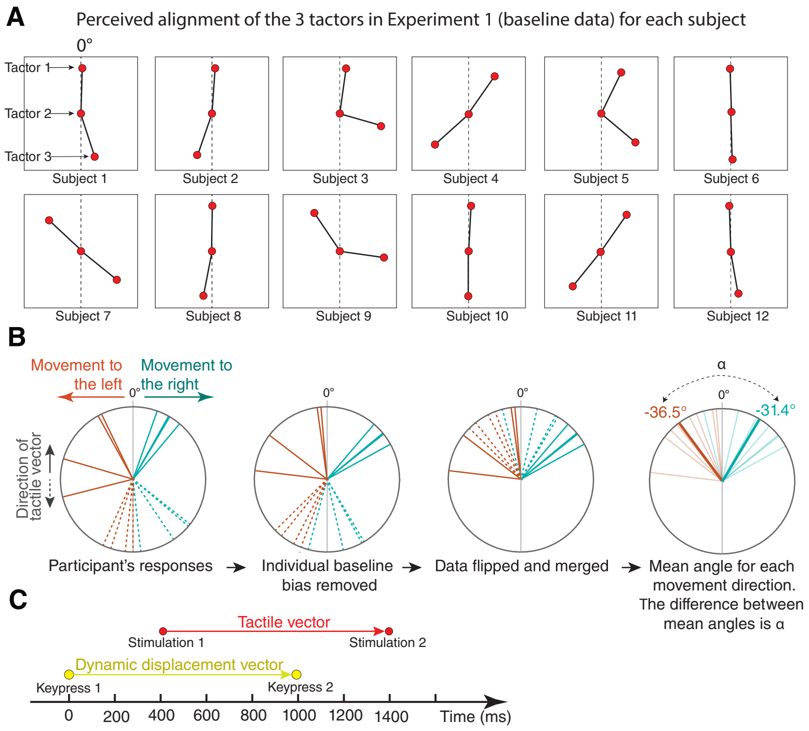

Figure S1. Baseline, steps of analysis and temporal description of one trial, related to STAR Methods. (A) Relative perceived position of the three motors of Experiment 1 computed from baseline data (without movement). Each square correspond to one participant. (B) Steps of data analysis to calculate the angle α for one participant of Experiment 1, active movement condition. The α value is the main dependent variable for this study and corresponds to the difference between mean angles of the tactile vectors for leftward and for rightward finger displacements. (C) Temporal description of one trial in Experiment 6 with a delay of 400 ms between tactile and displacement vector.

Exp.	Condition	Mean (s)	S.D
1	*Perpendicular-active*	1.11	0.25
	Perpendicular-passive	1.10	0.09
	Parallel-active	1.12	0.26
	Parallel-passive	1.11	0.12
2	*Same-limb*	1.00	0.08
	Different-limbs	1.05	0.1
	Finger-Forehead	1.04	0.1
3	*Standard*	1.12	0.27
	Sliding	1.21	0.3
	2-Fingers	0.99	0.17
4	*Perpendicular-Right*	0.99	0.19
	Parallel-Right	1.07	0.28
5	*V-Configuration*	0.96	0.17
6	*(All delays)*	1.0	0.08

Table S1. Movement durations, related to STAR Methods. Means and standard deviations of movements' durations over participants by condition. Movements were produced by the participant or the experimenter depending on condition. The instructed duration was 1 s.

Exp.	Condition	α predicted by vector summation with equal weighting (deg)	Observed α (deg)	Ratio Observed /Predicted	Equivalent finger displacement corresponding to observed α (cm)
1	*Perpendicular-active*	146.6	50.8	0.35	0.57
	Perpendicular-passive	146.6	82.3	0.56	1.05
2	*Same-limb*	146.6	71.6	0.49	0.87
	Different-limbs	146.6	72.3	0.49	0.88
	Finger-Forehead	146.6	45.2	0.31	0.5
3	*Standard*	146.6	62.7	0.43	0.73
	Sliding	146.6	95	0.65	1.31
	2-Fingers	146.6	50.9	0.35	0.57
4	*Perpendicular-Right*	146.6	51.1	0.35	0.57
5	*V-Configuration*	106.8	13.45	0.13	0.37

Table S2. Contribution of finger displacement vector to tactile orientation perception, related to STAR Methods. For each experiment and each condition (except parallel conditions), we calculated the hypothetical perceived orientation of the tactile vector, under the assumption of a simple vector summation between tactile and displacement vectors, with both vectors having equal weighting ("predicted orientation"). The biasing effect α observed in each condition of each experiment is shown, and is expressed as a proportion of the predicted orientation. Finally, we report the equivalent finger displacement that would be required to produce the observed α under the prediction of simple vector summation. This value provides an estimate of the influence of finger displacement on touch. The actual finger displacement was always 4 cm.
