# Supplementary material for: Dynamic Displacement Vector Interacts with Tactile Localization
Source: Curr Biol. 2019 Feb 4;29(3):492–498.e3. doi: 10.1016/j.cub.2018.12.032 (PMC6370943; doi:10.1016/j.cub.2018.12.032)
Supplement: Document S2. Article plus Supplemental Information [file mmc3.pdf]

Current Biology

Report

Dynamic Displacement Vector Interacts with Tactile Localization

Highlights

- Human tactile localization is biased by simultaneous finger displacement
- The shift between two successive events biases the relative localization of touches
- Both active and passive movements induce a bias, even if far from the touched site
- The bias effect is vectorially organized

Authors

Lucile Dupin, Patrick Haggard

Correspondence

lucile.dupin@parisdescartes.fr

In Brief

Perceiving the location of tactile stimuli is subject to important biases. Dupin and Haggard reveal that the displacement between two successive spatial events biases tactile localization on a remote body part. The building blocks of such spatial interactions are dynamic vectors between relative locations rather than snapshots of absolute location.

Check for updates

Dupin & Haggard, 2019, Current Biology *29*, 492–498
February 4, 2019 © 2018 The Author(s). Published by Elsevier Ltd.
https://doi.org/10.1016/j.cub.2018.12.032

CellPress

CellPress

Current Biology
Report

Dynamic Displacement Vector Interacts with Tactile Localization

Lucile Dupin,[1,2,*] and Patrick Haggard[1]
[1]Institute of Cognitive Neuroscience, University College London, London WC1N 3AR, UK
[2]Lead Contact
*Correspondence: lucile.dupin@parisdescartes.fr
https://doi.org/10.1016/j.cub.2018.12.032

SUMMARY

Locating a tactile stimulus on the body seems effortless and straightforward. However, the perceived location of a tactile stimulation can differ from its physical location [1–3]. Tactile mislocalizations can depend on the timing of successive stimulations [2, 4, 5], tactile motion mechanisms [6], or processes that "remap" stimuli from skin locations to external space coordinates [7–11]. We report six experiments demonstrating that the perception of tactile localization on a static body part is strongly affected by the displacement between the locations of two successive task-irrelevant actions. Participants moved their index finger between two keys. Each keypress triggered synchronous tactile stimulation at a randomized location on the immobilized wrist or forehead. Participants reported the location of the second tactile stimulation relative to the first. The direction of either active finger movements or passive finger displacements biased participants' tactile orientation judgements (experiment 1). The effect generalized to tactile stimuli delivered to other body sites (experiment 2). Two successive keypresses, by different fingers at distinct locations, reproduced the effect (experiment 3). The effect remained even when the hand that moved was placed far from the tactile stimulation site (experiments 4 and 5). Temporal synchrony within 600 ms between the movement and tactile stimulations was necessary for the effect (experiment 6). Our results indicate that a dynamic displacement vector, defined as the location of one sensorimotor event *relative* to the one before, plays a strong role in structuring tactile spatial perception.

RESULTS

Any body movement produces concomitant cutaneous sensations, so movement and tactile stimulation are inextricably linked [12, 13]. However, it remains unclear whether and how movement information affects tactile perception on body parts that do not move. In the present study, we hypothesized that a non-informative movement of one body part—the index finger—could change the perceived localization of tactile stimulations on an immobile body part. Such a finding would imply a dynamic reorganization of tactile perception by other sensorimotor inputs, possibly reflecting a supramodal attention mechanism in spatial perception.

We applied two successive tactile stimulations, defining a "tactile vector," on the immobile left wrist or the forehead (see Figures 1A and 1B and Video S1) while the participant moved their index finger to press two keys in succession. Pressing on each response key caused a tap from one of three tactile stimulators strapped to the wrist (or forehead). The location of the first key and their associated tap location were randomized, so that the displacement vector between the keypresses and the tactile vector between the two stimulations could be in either direction (Figure 1C). The instructed delay between the two taps was 1 s in order to prevent apparent motion effects [14]. Participants adjusted a pointer to indicate the perceived direction of the second tap location relative to the first (Figures 1A and 1D).

We hypothesized that pressing the key on the right at the time of the second tactile stimulation, though irrelevant to the tactile task, would cause a rightward tactile mislocalization, compared to the first tactile stimulation. This would produce a clockwise (positive) response direction (Figure 1D; Video S1; vice versa for a second left key). A significant difference between the angles for rightward versus leftward movements implies that the perceived tactile vector direction is influenced by the direction of the displacement.

Experiment 1: The Effects of Movement Direction and Passive versus Active Movement

This experiment tested whether task-irrelevant finger movements could interfere with tactile localization. The left index finger moved between two keypresses, taking 1 s. Two tactile stimulations occurred invisibly on the left wrist dorsum, synchronized with the two keypress events. The data of the two tactile vector directions were pooled following a flip-alignment procedure (see data analysis in STAR Methods and Figure S1B). There were two factors in the experimental design: the spatial orientation of the finger movement (orthogonal or parallel to the tactile vector; see Figures 1B and 1E, respectively), and how the movement was performed (actively or passively; see STAR Methods).

Perpendicular Displacement Conditions

For each participant, we calculated the mean perceived angle for both directions of the displacement vector: when the movement was from the left key (key 1) to the right key (key 2; Figure 1B), and for the opposite direction. The cumulated angular

 © 2018 The Author(s). Published by Elsevier Ltd.
This is an open access article under the CC BY license (http://creativecommons.org/licenses/by/4.0/).

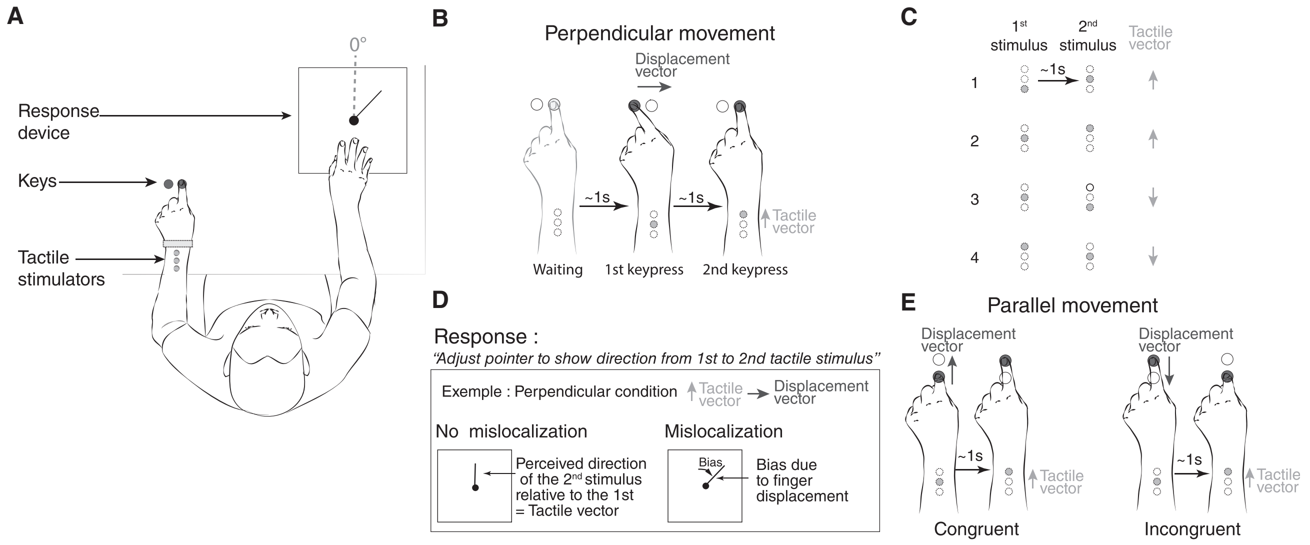

Figure 1. Experimental Setup, Task, and Conditions of Experiment 1

(A) Overview of the experimental setup.

(B) Example of one trial in the perpendicular condition of experiment 1. Each trial comprised 3 stages: a waiting foreperiod, followed by pressing on a first, and then a second, key. The movement between the first and second key is here to the right (could also be to the left). Each keypress triggers touch from one of three tactors on the wrist. The movement was therefore associated with a tactile vector (light gray) in the distal direction (could also be the proximal direction).

(C) Tactile stimuli define a tactile vector.

(D) Participants used a pointer to indicate the direction of a vector linking the tactile locations. Note the possibility of bias by finger displacement.

(E) Arrangement of the parallel movement condition of experiment 1. Note that the displacement vector and tactile vector can be congruent or incongruent. The figure illustrates one tactile vector direction that could also be in the opposite direction.

difference (α) between leftward- and rightward-biasing effects of finger movement on tactile localization was computed.

Results are illustrated in Figure 3A. The angle α was significantly different from 0 for both active (mean 50.8°, t_{11} = 3.24, p = 0.008) and passive conditions (mean 82.3°, t_{11} = 5.55, $p < 0.001$, t test), and was greater for passive than for active (t_{11} = 2.53, p = 0.03, paired t test).

Parallel Displacement Conditions

We sorted pointer responses into two categories: correct and inverted perception of the tactile vector. We compared the percentage of inversions when the tactile and displacement vectors pointed in the same direction (congruent) or opposite directions (incongruent; see Figure 1E). The percentage of inversions was 14.6% (SD 18.3, active movements) and 7.3% (SD 8.35, passive movements) in the congruent condition and 32.3% (SD 26.4, active movements) and 39.6% (SD 24.3, passive movements) in the incongruent condition. Inversions were more frequent in the incongruent condition (active, p = 0.031; passive, p = 0.039; sign tests are due to the non-normality of the congruent percentage of inversions).

Experiment 2: Locations of the Movement and Tactile Stimulations over the Body

We investigated whether the finger displacement vector could bias the tactile vector at body sites far from the moving finger, as well as nearby on the wrist. The design was based on the passive movement and perpendicular conditions of experiment 1. Tactile vectors were applied in three blocked conditions to the left wrist (Figure 2A): while the left index finger was passively displaced (same-limb condition, identical to experiment 1), to the left wrist while the right index finger was passively moved (different-limbs condition), and to the forehead while the left index finger was passively moved (finger-forehead condition).

Results are illustrated in Figure 3B. The angle α was significantly different from 0 in all 3 conditions: same-limb (mean 71.6°, t_9 = 4.52, p = 0.001), different-limbs (mean 72.3°, t_9 = 3.87, p = 0.004), and finger-forehead (mean 45.3°, p = 0.002, sign test due to non-normality). These results show mislocalization of touch was no greater on body sites close to the moving finger than on those farther away (all $p > 0.11$, paired t testing and sign tests between conditions).

Experiment 3: Different Types of Displacement

Experiment 3 investigated whether two specific features of finger movement could bias tactile localization. The first condition was similar to the active perpendicular condition of experiment 1 (standard condition). In the sliding condition, participants slid the finger across the surface of a card positioned between the two keys, giving continuous stimulation to the index fingertip (Figure 2B). If the biasing effect on tactile localization were due merely to spatial attraction between simultaneous keypress and tactile input, then this condition should blur and render indistinct the separate keypress events, weakening any attraction. In the 2-Fingers condition, participants positioned their index and middle fingers over the two keys and pressed them in order, without any mediolateral finger movement (Figure 2B). This condition will show whether movement between the two keys is necessary for biasing tactile localization.

CellPress

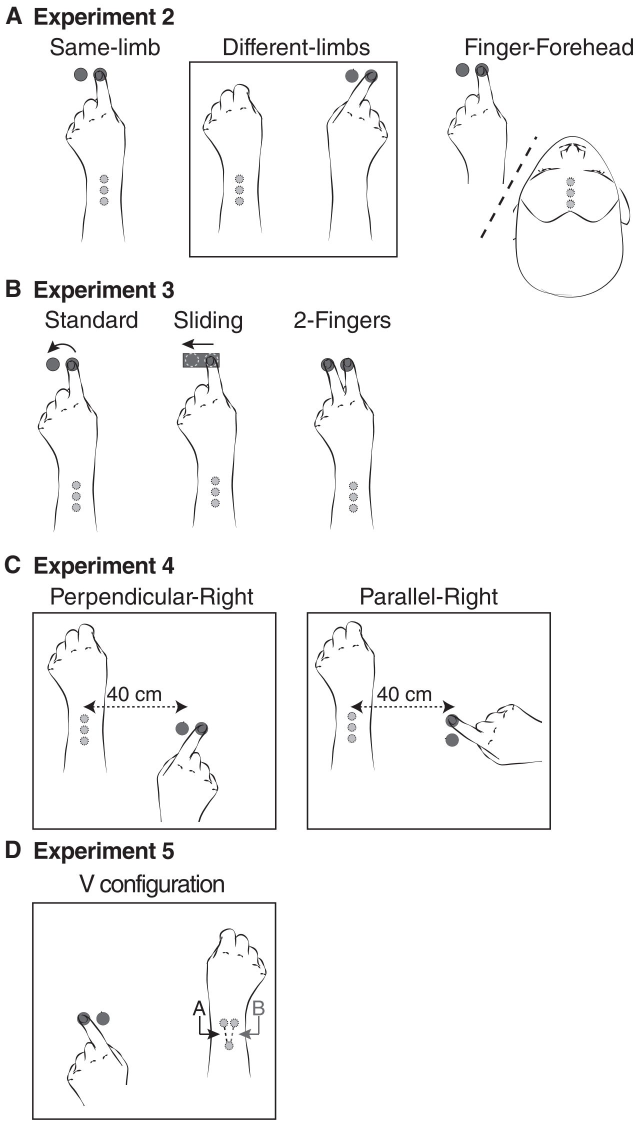

Figure 2. Conditions of Experiments 2–6

(A) Positions of the tactile stimulators and the two keys in experiment 2. The same-limb condition replicates the perpendicular condition of experiment 1. Different-limbs condition: the movement is produced by the right hand, and stimulations occur on the left wrist. Finger-forehead condition: the movement is produced by the left hand, and tactile stimuli are delivered to the forehead.

(B) Three movement conditions were compared in experiment 3: standard finger movement as in experiment 1, sliding movement along the table surface, and successive pressure on the two keys by two different fingers.

(C) Positions of tactile stimuli and keys for the perpendicular-right and parallel-right conditions of experiment 4 were based on experiment 1, except that the movement is produced by the right hand whereas touch stimuli are delivered to the left wrist. Note that the right hand's movement in the parallel condition of experiment 4 is similar to the left hand's movement in the perpendicular experiment of experiment 1.

(D) Positions of tactile stimuli in a V configuration and keys for experiment 5.

Results are illustrated in Figure 3C. For all conditions, α was significantly different from 0 (standard: 62.5°, t_9 = 3.5, p = 0.007; sliding: 95°, t_9 = 4.4, p = 0.002; 2-Fingers: 50.9°, t_9 = 3.9, p = 0.004). The finger displacement vector could influence tactile localization even without two distinct tactile stimulations (sliding condition) or the lateral movement of the finger (2-Fingers condition).

Experiment 4: Attraction to the Absolute or Relative Keypress Locations

This experiment aimed to distinguish whether the mislocalization bias was due to an interaction between absolute locations of keypresses and corresponding tactile locations, or to the relative position of one keypress with respect to another (i.e., a vector). Tactile stimulations were located on the left wrist and active keypresses were made with the right hand (cf. experiment 2). Whereas the keypress locations in experiment 1 were symmetrical around the tactile locations, here the keypress locations were offset 40 cm to one side and arranged in two possible movement orientations, perpendicular-right and parallel-right (Figure 2C). Previous studies have shown that spatial attraction of touch by vision [15] or by movement [16, 17] depends on spatial congruency and distance. The attraction decreases as the distance increases, with a sharp decrease beyond 30 cm [16]. Attraction between absolute individual locations predicts a decrease of the bias compared to experiment 1, whereas a dynamic displacement vector hypothesis would predict similar results to experiment 1.

Perpendicular Displacement Condition

Results are illustrated in Figure 3D. The mean biasing angle of 54.1° was significantly different from 0 (t_9 = 2.94, p = 0.02, t test). A between-experiment comparison showed no significant difference with the perpendicular active condition of experiment 1 (mean 50.8°, t_{20} = 0.14, p = 0.89).

Parallel Displacement Condition

The percentage of tactile vectors perceived as inverted (cf. experiment 1) was 2.5% (SD 5.3) in the congruent condition (not significantly different from 0, p = 0.5, sign test) and 30% (SD 32.4, significantly different from 0, p = 0.004, sign test) in the incongruent condition. These values differed significantly (p = 0.016, paired sign test). However, inversions in the incongruent condition were no more frequent than in the active movement condition of experiment 1 (p = 0.85, t test), despite the large difference in the distance between touch and movement in the two experiments. Thus, the movement-touch interaction occurs at the level of vectors between relative positions, not at the level of absolute spatial locations.

Experiment 5: Geometric Principle of Tactile Vector Combination

In experiments 1–4, the collinear configuration of the tactile stimulators allowed only one orientation of the tactile vector, though two directions were presented. The invariant stimulus orientation causes difficulty in separating perceptual sensitivity of reported orientation from bias—although we have no particular evidence that strong bias occurred. Therefore, experiment 5 used two different stimulus orientations, each again presented in two directions. This configuration allows the sensitivity and bias components of participants' responses to be identified. The setup of this experiment was broadly similar to the perpendicular-right condition of experiment 4, except that finger movements were

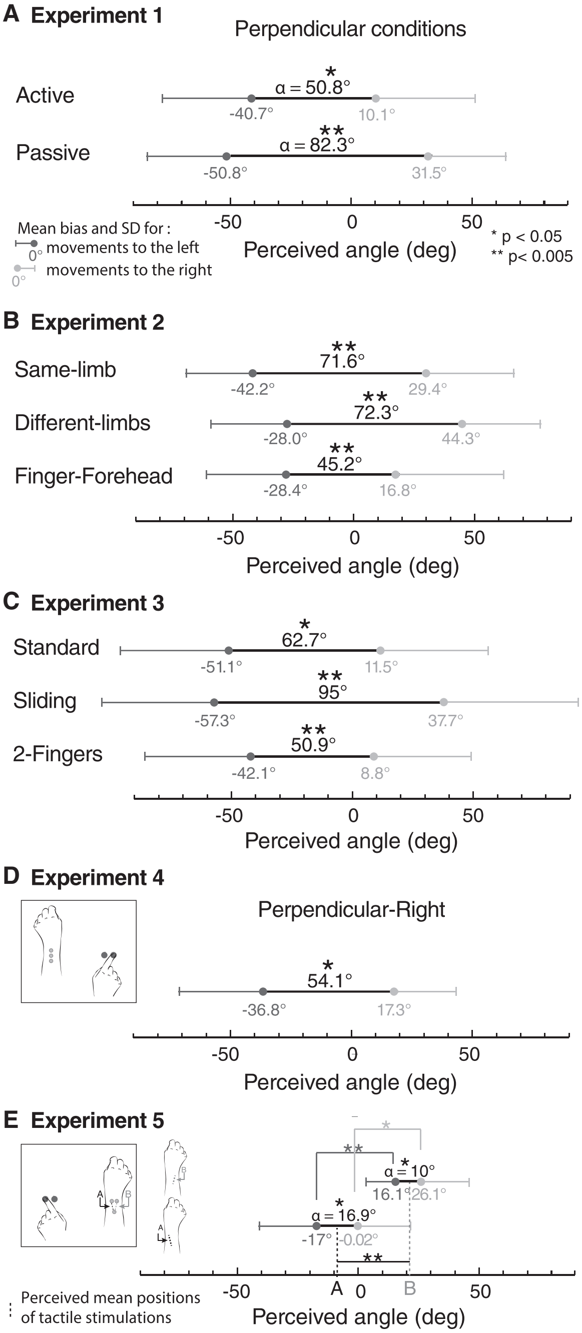

Figure 3. Effect of Finger Movement Direction Experiments 1–5

(A) Grand mean and SD across participants of the perceived tactile direction angle in perpendicular conditions during displacements to the left (dark gray) or right (light gray), while making active and passive movements. The total angle α (black line) indicates the biasing effect of finger displacement on tactile direction perception.

(B) Movement-induced bias in tactile vectors for the three conditions of experiment 2: same-limb, different-limbs, and finger-forehead.

(C) Movement-induced bias in tactile vectors for the three conditions of experiment 3: standard movement, sliding movement, and 2-Fingers.

(D) Experiment 4: effects of moving the right finger on left wrist tactile perception for the perpendicular-right movement condition.

(E) Experiment 5: effects of moving the left finger on right wrist tactile perception in a V configuration.

made with the left hand, whereas touch was delivered to the right wrist. Further, the tactors were configured in a V shape, and the length of the tactile vector was increased to 3 cm. Thus, there were two possible tactile vector orientations for the tactile vector (marked A and B, Figure 2D), combined with two directions of finger movement. Results are shown in Figure 3E. ANOVA found a main effect of finger movement direction ($F_{1,9}$ = 15.43, p = 0.003), replicating the biasing effect of finger movement on tactile orientation, and a main effect of tactile vector orientation ($F_{1,9}$ = 9.17, p = 0.014), confirming participants' perceptual sensitivity to the stimulus orientation. Interestingly, we found no evidence for interaction between tactile stimulus orientation and finger movement direction ($F_{1,9}$ = 0.58, p = 0.47), suggesting that tactile stimulus orientation and finger movement may be independent and additive factors. Because the tactile vector of experiment 5 involved a greater spatial separation between tactile stimuli than previous experiments, we could test whether finger displacement biases tactile perception by a constant amount, or to an extent that reflects a geometric vector summation of the movement and tactile vectors. The latter account predicts that the α angle measure of bias should be smaller in experiment 5 than in the perpendicular-right condition of experiment 4. This prediction was confirmed (p = 0.02, sign test).

Interestingly, the perceived tactile vectors in Figure 3E showed a slight overall rightward bias (t_9 = 3.02, p = 0.014, one-sample test against 0), in contrast to the slight overall leftward bias in other experiments. Comparison with the experiment 4 perpendicular-right condition showed that these global shifts differed between experiments (t_9 = 3.02, p = 0.014, unpaired t test). We speculate that these global shifts reflect differences between the experiments in the assignment of touch and finger movement to the two hands, and the hands' positions in egocentric space (Figures 2C and 2D). Haptic perception of parallelism varies systematically according to both the limb stimulated and to its position in egocentric space [18]. Inverting the role of the two hands in experiment 5 relative to experiment 4 thus shifted the perceived tactile vector toward a rightward (clockwise) orientation.

Experiment 6: Temporal Shift between Movement and Tactile Stimulation

The aim of experiment 6 was to identify whether synchrony between keypress and tactile stimulation was a necessary condition for the observation of a localization bias.

Here we added a delay of 0, 200, 400, 600, or 800 ms between each keypress and the corresponding tactile stimulation to the passive and perpendicular condition of experiment 1 (see Figure S1C for an example). We envisaged three possible effects of delay on tactile mislocalization. First, the dynamic displacement vector hypothesis predicts no effect of delay on mislocalization, because the vector between successive finger events, and the vector between successive tactile events, are both unaffected. Alternatively, a hypothesis of spatial attraction between each individual finger tactile event and the associated tactile event predicts that such delays should abolish the mislocalization, by weakening the association between each finger event and corresponding tactile event. Third, one might hypothesize that each tactile onset triggers sampling of the finger position. Because the finger remained immobile on the second key, the

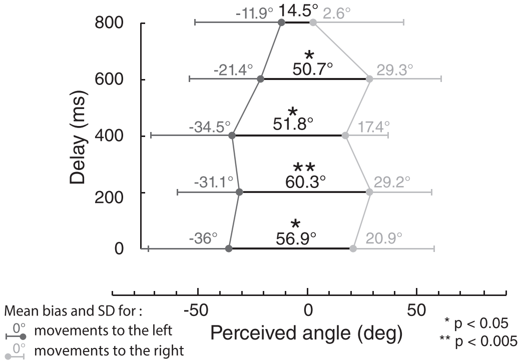

Figure 4. Effect of Finger Movement Direction in Function of the Asynchrony Delay in Experiment 6

Effects of a movement-tactile asynchrony in experiment 6, using the same setup as the experiment 1 passive movement, perpendicular condition; notice the persistence of movement-induced bias of tactile direction judgement for delays up to 600 ms.

change in finger position between the first and second tactile event will decrease with delay, implying a gradually decreasing bias.

Results are illustrated in Figure 4. Repeated-measures ANOVA showed a significant effect of the delay on the bias angle ($F_{1,9}$ = 3.2, p = 0.02, partial η^2 = 0.26). The α values showed significant bias from 0 ms to 600 ms (t_9 = 3.4, p = 0.008; t_9 = 4, p = 0.003; t_9 = 3.3, p = 0.01; t_9 = 3.1, p = 0.012) but not for 800 ms (t_9 = 1.16, p = 0.28). Paired comparisons did not show any significance between delays (p > 0.15 for all comparisons, Bonferroni corrected). The results support the dynamic displacement vector hypothesis, at least up to 600 ms.

DISCUSSION

We showed that a displacement vector linking finger event positions interacts with tactile localization. An effect of finger keypress locations on tactile direction judgements was found in both passive and active movement conditions (experiment 1), suggesting active motor commands are not necessary. The biasing of the tactile vector by the displacement vector operates across distant body sites (experiment 2), across multiple different orientations of tactile stimulation (experiment 5), over large spatial separations between displacement vector and tactile vector, and even interhemispherically (experiments 2 and 4). Across all experiments, we found that the angle α was always less than a perfect geometric summation of finger and tactile vectors would predict, implying that the contribution of finger displacement to tactile perception has a rather low weighting. However, comparison of different tactile stimulation configurations (e.g., experiment 4 versus 5) confirmed that the bias follows a general geometric principle of vector summation, though the weighting of the finger and tactile vectors may vary (experiment 5). The finger displacement vector is not simply the representation of an aimed *movement* between these locations, as separate keypress movements at two locations are sufficient for the effect (experiment 3). The finger displacement vector remains available for association with the tactile vector for up to 600 ms (experiment 6). Studies have emphasized remapping of skin input to external spatial locations, typically using uncommon postures (crossed arms and fingers) [9, 10, 19] or localization on moving body parts [20]. Remapping is normally considered to occur automatically [21] and within 70 ms of stimulation [22], suggesting spatiotopic representations dominate somatotopic representations. We consistently found that spatiotopic relation between the finger displacement vector and the tactile vector played a key role in tactile localization. A fixed spatial transformation process from the somatotopic reference frame to external space might introduce biases in absolute tactile localization. Importantly, *relative* localization of nearby tactile stimuli should be unaffected by such transformations. However, the spatial transformation may differ between two successive tactile stimulations because of the automatic integration of a concurrent displacement vector, even when the displacement is irrelevant and spatially remote [13, 23]. In our case, the two successive tactile stimuli would use different remapping transformations, producing biases even in relative localization judgement.

We have shown that the bias is not due to the movement of the finger per se, because it occurs even when two different fingers are used to press the two response keys. We found that the effect of finger displacement on tactile vector orientation varied in size across experiments. In experiments 1–4, tactile stimuli involved only one single orientation (though direction was varied). Experiment 5 confirmed that orientation judgements did indeed covary with stimulus orientation, demonstrating perceptual sensitivity—and a highly significant effect of finger displacement was again found. Nevertheless, the effect in experiment 5 was smaller than in experiment 4. The difference in effect size may reflect the different geometries of tactile stimulation in the two experiments (see Figures 2C and 2D and Table S2), or a difference in some other factor such as response bias. However, the effect of finger displacement was consistently present.

Similarly, the effect of finger displacement on tactile perception is not motoric in origin, because it was present also when the finger was moved passively. Instead, we propose that the underlying mechanism could be related to the shifting of attention from the location of the first to the second keypress event. In the context of remapping theories [21, 24], shifting attention to a new location could influence the transformation from skin locations to external locations, leading to relative mislocalization in the same direction as the shift of attention. Critically, this account is based on conceiving attention shift as a vector between successive locations, rather than a spotlight on a single, current location.

Saccadic eye movements produce systematic mislocalization of stimuli displayed around the saccade onset toward the saccade target [25–28] that could be related to associated attentional shifts [29–31]. Moreover, visual spatial attention [32], saccades, or gaze shifts have been shown to bias tactile localization judgement [32–34]. Importantly, gaze shifts are necessary to update the tactile spatial localization in a gaze-centered reference frame [33]. Consequently, tactile mislocalizations reported here could depend on *shifts* of attention, from one location to another, in line with supramodal accounts of attention [35, 36].

We conclude that any sensorimotor sequence of events at two distinct spatial locations defines a dynamic displacement vector that biases the spatial perception of co-occurring tactile

stimulation. Our results progress discussions of somatosensory spatial attention away from the previous focus on coding of *locations*, and toward shifts of attention that define a *vector* linking the location of one event to the location of the next. We speculate that the brain's representation of external space may lack any truly fixed point of origin. Rather, each new event may reset the effective point of origin, so that we continually perceive a vector that relates the location of one stimulus to the location of the next. Our results suggest that computing this vector for touch involves an automatic integration of finger displacement information. This implies a common, amodal code for these relative position vectors.

STAR★METHODS

Detailed methods are provided in the online version of this paper and include the following:

- KEY RESOURCES TABLE
- CONTACT FOR REAGENT AND RESOURCE SHARING
- EXPERIMENTAL MODEL AND SUBJECT DETAILS
- METHOD DETAILS
  - Apparatus
  - Condition parameters
  - Procedure
  - Baseline
- QUANTIFICATION AND STATISTICAL ANALYSIS
- DATA AND SOFTWARE AVAILABILITY

SUPPLEMENTAL INFORMATION

Supplemental Information includes one figure, two tables, and one video and can be found with this article online at https://doi.org/10.1016/j.cub.2018.12.032.

ACKNOWLEDGMENTS

We thank Vincent Hayward and Antonio Cataldo for advice and assistance. This study was supported by a post-doctoral fellowship from the Fondation Fyssen (to L.D.). Additional support was provided by ERC advanced grant HUMVOL (agreement number 323943) and MRC project grant MR/M013901/1 (to P.H.).

AUTHOR CONTRIBUTIONS

Conceptualization, Methodology, & Writing, L.D. and P.H.; Software, Investigation, Formal Analysis, & Visualization, L.D.; Supervision, P.H.

DECLARATION OF INTERESTS

The authors declare no competing interests.

Received: June 24, 2018
Revised: November 15, 2018
Accepted: December 18, 2018
Published: January 24, 2019

REFERENCES

1. Hayward, V. (2008). A brief taxonomy of tactile illusions and demonstrations that can be done in a hardware store. Brain Res. Bull. *75*, 742–752.
2. Geldard, F.A. (1985). The mutability of time and space on the skin. J. Acoust. Soc. Am. *77*, 233–237.
3. Culver, C.M. (1970). Errors in tactile localization. Am. J. Psychol. *83*, 420–427.
4. Jones, F.N. (1956). Space-time relationships in somesthetic localization. Science *124*, 484.
5. Sadibolova, R., Tamè, L., Walsh, E., and Longo, M.R. (2018). Mind the gap: the effects of temporal and spatial separation in localization of dual touches on the hand. Front. Hum. Neurosci. *12*, 55.
6. Seizova-Cajic, T., and Taylor, J.L. (2014). Somatosensory space abridged: rapid change in tactile localization using a motion stimulus. PLoS ONE *9*, e90892.
7. Heed, T., Buchholz, V.N., Engel, A.K., and Röder, B. (2015). Tactile remapping: from coordinate transformation to integration in sensorimotor processing. Trends Cogn. Sci. *19*, 251–258.
8. Benedetti, F. (1988). Localization of tactile stimuli and body parts in space: two dissociated perceptual experiences revealed by a lack of constancy in the presence of position sense and motor activity. J. Exp. Psychol. Hum. Percept. Perform. *14*, 69–76.
9. Longo, M.R., Mancini, F., and Haggard, P. (2015). Implicit body representations and tactile spatial remapping. Acta Psychol. (Amst.) *160*, 77–87.
10. Azañón, E., Stenner, M.-P., Cardini, F., and Haggard, P. (2015). Dynamic tuning of tactile localization to body posture. Curr. Biol. *25*, 512–517.
11. Watanabe, J., Nakatani, M., Ando, H., and Tachi, S. (2009). Haptic localizations for onset and offset of vibro-tactile stimuli are dissociated. Exp. Brain Res. *193*, 483–489.
12. Dupin, L., Hayward, V., and Wexler, M. (2015). Direct coupling of haptic signals between hands. Proc. Natl. Acad. Sci. USA *112*, 619–624.
13. Dupin, L., Hayward, V., and Wexler, M. (2017). Generalized movement representation in haptic perception. J. Exp. Psychol. Hum. Percept. Perform. *43*, 581–595.
14. Kirman, J.H. (1974). Tactile apparent movement: the effects of interstimulus onset interval and stimulus duration. Percept. Psychophys. *15*, 1–6.
15. Pavani, F., Spence, C., and Driver, J. (2000). Visual capture of touch: out-of-the-body experiences with rubber gloves. Psychol. Sci. *11*, 353–359.
16. Lloyd, D.M. (2007). Spatial limits on referred touch to an alien limb may reflect boundaries of visuo-tactile peripersonal space surrounding the hand. Brain Cogn. *64*, 104–109.
17. Aimola Davies, A.M., White, R.C., and Davies, M. (2013). Spatial limits on the nonvisual self-touch illusion and the visual rubber hand illusion: subjective experience of the illusion and proprioceptive drift. Conscious. Cogn. *22*, 613–636.
18. Kappers, A.M.L. (1999). Large systematic deviations in the haptic perception of parallelity. Perception *28*, 1001–1012.
19. Heed, T., Backhaus, J., and Röder, B. (2012). Integration of hand and finger location in external spatial coordinates for tactile localization. J. Exp. Psychol. Hum. Percept. Perform. *38*, 386–401.
20. Maij, F., Wing, A.M., and Medendorp, W.P. (2013). Spatiotemporal integration for tactile localization during arm movements: a probabilistic approach. J. Neurophysiol. *110*, 2661–2669.
21. Azañón, E., Camacho, K., and Soto-Faraco, S. (2010). Tactile remapping beyond space. Eur. J. Neurosci. *31*, 1858–1867.
22. Soto-Faraco, S., and Azañón, E. (2013). Electrophysiological correlates of tactile remapping. Neuropsychologia *51*, 1584–1594.
23. Dupin, L., Hayward, V., and Wexler, M. (2018). Radial trunk-centred reference frame in haptic perception. Sci. Rep. *8*, 13550.
24. Overvliet, K.E., Azañón, E., and Soto-Faraco, S. (2011). Somatosensory saccades reveal the timing of tactile spatial remapping. Neuropsychologia *49*, 3046–3052.
25. Szinte, M., Wexler, M., and Cavanagh, P. (2012). Temporal dynamics of remapping captured by peri-saccadic continuous motion. J. Vis. *12*, 12.
26. Kaiser, M., and Lappe, M. (2004). Perisaccadic mislocalization orthogonal to saccade direction. Neuron *41*, 293–300.

27. Honda, H. (1989). Perceptual localization of visual stimuli flashed during saccades. Percept. Psychophys. *45*, 162–174.
28. Ross, J., Morrone, M.C., Goldberg, M.E., and Burr, D.C. (2001). Changes in visual perception at the time of saccades. Trends Neurosci. *24*, 113–121.
29. Shim, W.M., and Cavanagh, P. (2006). Bi-directional illusory position shifts toward the end point of apparent motion. Vision Res. *46*, 3214–3222.
30. Shim, W.M., and Cavanagh, P. (2005). Attentive tracking shifts the perceived location of a nearby flash. Vision Res. *45*, 3253–3261.
31. Hoffman, J.E., and Subramaniam, B. (1995). The role of visual attention in saccadic eye movements. Percept. Psychophys. *57*, 787–795.
32. Harrar, V., and Harris, L.R. (2009). Eye position affects the perceived location of touch. Exp. Brain Res. *198*, 403–410.
33. Mueller, S., and Fiehler, K. (2014). Gaze-dependent spatial updating of tactile targets in a localization task. Front. Psychol. *5*, 66.
34. Pritchett, L.M., and Harris, L.R. (2011). Perceived touch location is coded using a gaze signal. Exp. Brain Res. *213*, 229–234.
35. Macaluso, E., Frith, C.D., and Driver, J. (2002). Supramodal effects of covert spatial orienting triggered by visual or tactile events. J. Cogn. Neurosci. *14*, 389–401.
36. Macaluso, E., and Driver, J. (2001). Spatial attention and crossmodal interactions between vision and touch. Neuropsychologia *39*, 1304–1316.

STAR★METHODS

KEY RESOURCES TABLE

REAGENT or RESOURCE	SOURCE	IDENTIFIER
Software and Algorithms		
Mathematica 10.1	Wolfram	https://www.wolfram.com/mathematica/
SPSS Statistic 23	IBM	https://www.ibm.com/analytics/
Arduino	Arduino	https://www.arduino.cc/
Visual C++ Express 2010	Microsoft	https://www.visualstudio.com/
Deposited Data		
Individual mean angles or inversions for each experiment	Mendeley Data	https://doi.org/10.17632/67srggw35g.1
Other		
Linear actuator	Haydon Kerk	Motion Solutions 15000,Series LC1574W-04
Force-sensitive resistor	Interlink electronic	model 402
Potentiometer	ETI Systems	SP22G-5K
Arduino microcontroller	Arduino	Mega 2560 Rev3

CONTACT FOR REAGENT AND RESOURCE SHARING

Further information and requests for resources should be directed to and will be fulfilled by the Lead Contact, Lucile Dupin (Lucile.dupin@parisdescartes.fr).

EXPERIMENTAL MODEL AND SUBJECT DETAILS

All the participants were different between experiments. No individual participated in two experiments. All the participants were naive about the hypotheses of the experiment and were compensated £5. All the participants were self-told right-handed without history of hand injury or neurological disorders. The experimental protocol was approved by the research ethics committee of University College London. The study adhered to the ethical standards of the Declaration of Helsinki. All participants provided their written informed consent before the beginning of each experiment. The 62 different participants who took part in the six experiments were different.

Experiment 1: 12 participants (6 females, mean age 34.5, sd. 12.7 from 18 to 58 years old) took part in this experiment.
Experiment 2: 10 participants (4 females, mean age 28.5, sd. 8.2) took part in this experiment. One participant was excluded because he/she could not reach the baseline (the perception of the tactile vector orientations were not significantly different between distal to proximal and proximal to distal orientations in the baseline condition)
Experiment 3: 10 participants (7 females, mean age 21.5, sd. 1.8) took part in this experiment.
Experiment 4: 10 participants (7 females, mean age 23.6, sd. 4.1) took part in this experiment.
Experiment 5: 10 participants (5 females, mean age 35.1, sd. 7.8) took part in this experiment.
Experiment 6: 10 participants (8 females, mean age 27, ds. 9.5 from 19 to 49 years old) took part in this experiment

METHOD DETAILS

Apparatus

The apparatus was compounded by three devices (see Figure 1A for an overview) managed by a microcontroller (Arduino).

The first device was used to generate tactile stimulations. It was compounded by 3 stepper linear actuators (*Haydon Kerk Motion Solutions* 15000 series, model LC1574W-04). The linear motors generated pressure on a soft plastic surface positioned on the skin. The duration to the maximum pressure was 100ms and the linear displacement was 4 mm for Experiments 1 to 4. The duration to the maximum pressure was 80ms for Experiment 5 with the linear amplitude was 3.2 mm. The distance between 2 successive motors were approximately 1.2 cm for all experiments except Experiment 5 where the distance of motors A and B tactile was 3 cm with an angle between A and B of approximately 30° (see Figure 2D). The device could be attached

around the wrist to stimulate the dorsal side of the wrist (Experiments 1, 2, 4, 5, 6, see Figure 1A) or around the head to stimulate the forehead (Experiment 3, see Figure 2A)

The second device was used to drive the displacement. The two keys were two force sensors resistors (FSR Interlink model 402 FSR). The distance between the two force sensors was 4 cm. A force of 0.02 N applied on the force sensor was the threshold to launch a tactile stimulation. This device could be positioned at different locations to be used by the right or on the left.

The third device was used by the participant to respond. The main component was a continuous potentiometer (model SP22G-5K from ETI Systems). An arrow mounted on this potentiometer allows the participant to adjust the angle response corresponding to the orientation of the second tactile stimulation relative to the first one. This potentiometer was integrated in a square box of 20 cm side. This device was positioned in front of the participant on his/her right (Experiments 1-4 and 6) or on his/her left (Experiment 5).

Condition parameters

In Experiment 1 to 4, there were 16 trials for each condition. Each condition corresponded to one block. The parameters were the pair of motors selected in one trial (4 possibilities: 1-2, 2-3, 3-2, and 2-1 see Figure 1C) and the direction of movement (2 possibilities). Each combination was repeated 4 times. In Experiment 5, there were 4 trials for each direction of A and B tactile vectors (see Figure 2D) for one block, repeated one time.

In Experiment 6, there were three different parameters: the direction of the movement (2 possibilities), the delay between the key press and the delay of the tactile stimulation (5 possibilities, Figure S1C) and the pair of motors (4 possibilities). Each combination was repeated two times. The total number of trial was 80 divided in two different blocks.

In baseline conditions, there were 16 randomized trials with 4 repetitions of the 4 possible tactile vectors (2 pairs of tactors x 2 directions) illustrated in Figure 1C.

Procedure

In all conditions and experiments, participants were instructed to keep the eyes closed during the trial and the response.

Each trial started with an instruction indicating on which key the index finger of the participant has to be positioned. In all conditions the instruction was 'left' or 'right' except for the *Parallel* and *Parallel-Right* where the instruction was 'far' or 'close'. Then two "tick" sounds were played, separated by 1 s, to indicate the desired interval between the first and second keypress. The delay between the two tick sounds was an indication of timing for the participant movement (between two key presses).

The index finger of participant moved to the first key and the first tactile stimulation occurred, then he/she moved back to the second key and the second tactile stimulation occurred. The mean inter-keypress interval was 1.06 s (see Table S1 for details). Participants adjusted the orientation of a pointer to indicate the location of the second tactile stimulus relative to the first (see Figure 1D; Video S1). Then the experimenter pressed a key to launch the next trial.

In active movement conditions the movement was performed by the participant while in passive movement conditions the experimenter moved the index finger of the participant.

Baseline

In baseline conditions, each trial started with two tick sound separated by 1 s. After 1 s, two successive tactile stimulations occurred on the wrist or on the forehead (ISI = 1 s). Then the participant adjusted the arrow of response device to indicate the perceived angle between the first stimulation and the second stimulation. Finally, the experimenter pressed a key in order to launch the next trial. Individual perceived relative positions of the three motors for Experiment 1 computed from baseline data are presented in Figure S1A.

QUANTIFICATION AND STATISTICAL ANALYSIS

In order to standardize angle perception between participants, the mean angle obtained from the baseline condition was removed from the angles responded in displacement conditions. The mean angle for each movement direction or baseline was obtained using this formula:

$$\text{Mean } \alpha = Arctan\left(\frac{\sum_1^n \sin\alpha_i}{\sum_1^n \cos\alpha_i}\right)$$

Then, when tactile stimulations occurred on the wrist, we flipped all responses corresponding to a proximal second stimulus and merged them with data corresponding to distal stimulus. When the tactile stimulations were located on the forehead, the second stimulus could be upper or lower. Similarly we mirrored responses corresponding to a lower second stimulus and merged them with responses corresponding to an upper second stimulus. To mirror data, we inverted the y-coordinate of response angles α. Mirrored angles were then merge with angles corresponding to the initial opposite tactile vector orientation. All results are presented for one orientation of *tactile vector.* To each trial corresponded a response angle α_i. To compute the mean angle, we first calculate the polar coordinates of this angle: cos α_i and cos α_i. An example of the different steps on the analysis for one participant is illustrated in Figure S1B.

In Experiments 1 to 5, we analyzed data corresponding to movement durations that was lower than 2 s (the instructed duration was 1 s). In experiment 6, we analyzed data where movement durations were between 0.8 s and 1.2 s since the aim of this experiment

was to study the effect of delay. This excluded data represents 1.2% of the trials for Experiments 1 to 5 and 5.5% for Experiment 6. In Parallel conditions of Experiment 1 and 4, we used sign tests when non-normality of data was observed.

The contribution of finger displacement vector to tactile orientation perception for each experiment is detailed in Table S2.

DATA AND SOFTWARE AVAILABILITY

The file Angles.csv for individual mean angles or inversions for each experiment have been deposited into Mendeley Data at https://doi.org/10.17632/67srggw35g.1.

Current Biology, Volume *29*

Supplemental Information

Dynamic Displacement Vector Interacts with Tactile Localization

Lucile Dupin and Patrick Haggard

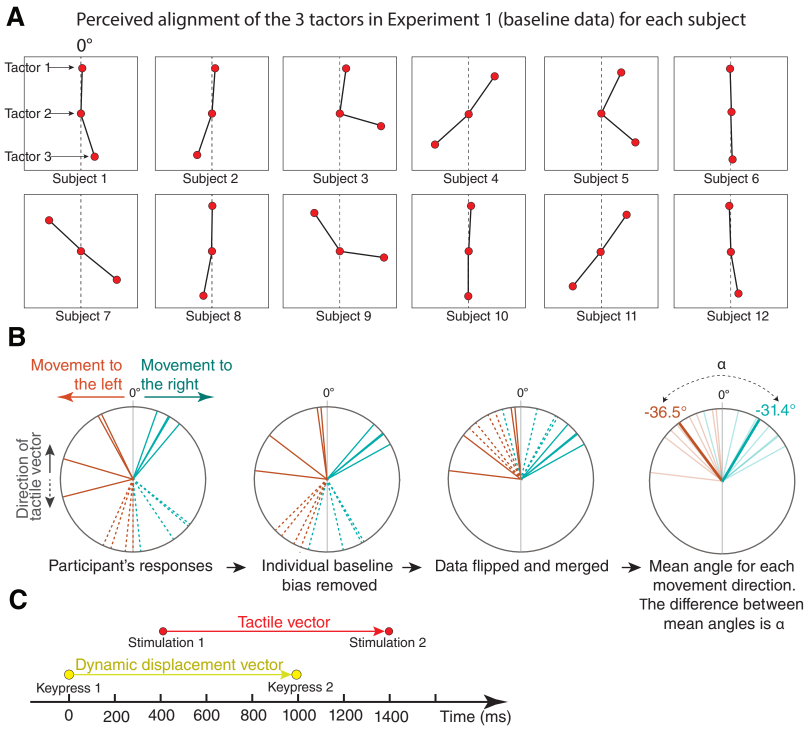

Figure S1. Baseline, steps of analysis and temporal description of one trial, related to STAR Methods. (A) Relative perceived position of the three motors of Experiment 1 computed from baseline data (without movement). Each square correspond to one participant. (B) Steps of data analysis to calculate the angle α for one participant of Experiment 1, active movement condition. The α value is the main dependent variable for this study and corresponds to the difference between mean angles of the tactile vectors for leftward and for rightward finger displacements. (C) Temporal description of one trial in Experiment 6 with a delay of 400 ms between tactile and displacement vector.

Exp.	Condition	Mean (s)	S.D
**1**	*Perpendicular-active*	1.11	0.25
	Perpendicular-passive	1.10	0.09
	Parallel-active	1.12	0.26
	Parallel-passive	1.11	0.12
**2**	*Same-limb*	1.00	0.08
	Different-limbs	1.05	0.1
	Finger-Forehead	1.04	0.1
**3**	*Standard*	1.12	0.27
	Sliding	1.21	0.3
	2-Fingers	0.99	0.17
**4**	*Perpendicular-Right*	0.99	0.19
	Parallel-Right	1.07	0.28
**5**	*V-Configuration*	0.96	0.17
**6**	*(All delays)*	1.0	0.08

Table S1. Movement durations, related to STAR Methods. Means and standard deviations of movements' durations over participants by condition. Movements were produced by the participant or the experimenter depending on condition. The instructed duration was 1 s.

Exp.	Condition	α predicted by vector summation with equal weighting (deg)	Observed α (deg)	Ratio Observed /Predicted	Equivalent finger displacement corresponding to observed α (cm)
**1**	*Perpendicular-active*	146.6	50.8	0.35	0.57
	Perpendicular-passive	146.6	82.3	0.56	1.05
**2**	*Same-limb*	146.6	71.6	0.49	0.87
	Different-limbs	146.6	72.3	0.49	0.88
	Finger-Forehead	146.6	45.2	0.31	0.5
**3**	*Standard*	146.6	62.7	0.43	0.73
	Sliding	146.6	95	0.65	1.31
	2-Fingers	146.6	50.9	0.35	0.57
**4**	*Perpendicular-Right*	146.6	51.1	0.35	0.57
**5**	*V-Configuration*	106.8	13.45	0.13	0.37

Table S2. Contribution of finger displacement vector to tactile orientation perception, related to STAR Methods. For each experiment and each condition (except parallel conditions), we calculated the hypothetical perceived orientation of the tactile vector, under the assumption of a simple vector summation between tactile and displacement vectors, with both vectors having equal weighting ("predicted orientation"). The biasing effect α observed in each condition of each experiment is shown, and is expressed as a proportion of the predicted orientation. Finally, we report the equivalent finger displacement that would be required to produce the observed α under the prediction of simple vector summation. This value provides an estimate of the influence of finger displacement on touch. The actual finger displacement was always 4 cm.
